# Association of change in health-related quality of life and treatment discontinuation in metastatic breast cancer: a post hoc, exploratory analysis of two randomized clinical trials

**DOI:** 10.1007/s00520-022-07283-0

**Published:** 2022-07-20

**Authors:** Takuya Kawahara, Takayuki Iwamoto, Ikumi Takashima, Ryoichi Hanazawa, Kohei Uemura, Yukari Uemura, Hirofumi Mukai, Yuichiro Kikawa, Naruto Taira

**Affiliations:** 1 Clinical Research Promotion Center, The University of Tokyo Hospital, 7-3-1, Hongo, Bunkyo-ku, Tokyo, 113-8655 Japan; 2 Departments of Breast and Endocrine Surgery, Okayama University Hospital, 2-5-1, Shikata-cho, Kita-ku, Okayama, 700-8558 Japan; 3 Department of Clinical Biostatistics, Graduate School of Medical and Dental Sciences, Tokyo Medical and Dental University, 1-5-45 Yushima, Bunkyo-ku, Tokyo, 113-8510 Japan; 4 Department of Biostatistics and Bioinformatics, Interfaculty Initiative in Information Studies, The University of Tokyo, 7-3-1 Hongo, Bunkyo-ku, Tokyo, 113-8654 Japan; 5 Department of Clinical Research, National Center for Global Health and Medicine, 1-21-1, Toyama, Shinjuku-ku, Tokyo, 162-8655 Japan; 6 Department of Breast and Medical Oncology, National Cancer Center Hospital East, 6-5-1, Kashiwa-city, Chiba, 277-8577 Japan; 7 Department of Breast Surgery, Kansai Medical University Hospital, 2-3-1, Hirakata, Osaka 573-0101 Japan; 8 Department of Breast and Thyroid Surgery, Kawasaki Medical School, 577 Matsushima, Kurashiki-city, Okayama, 701-0192 Japan

**Keywords:** Chemotherapy, Dose reduction, Metastatic breast cancer, Quality of life, Treatment discontinuation

## Abstract

**Purpose:**

Identifying factors associated with treatment alteration (treatment discontinuation and dose reduction) may help to attain the treatment goals for metastatic breast cancer. The value of changes in the quality of life (QOL) in predicting treatment alteration remained unclear. This study aimed to examine the relationship between changes in the QOL and treatment alteration of first-line chemotherapy for metastatic breast cancer.

**Methods:**

We merged data from two randomized clinical trials in Japan, conducted from 2006 to 2017, that included patients who were diagnosed with human epidermal growth factor receptor 2-negative and endocrine treatment-resistant breast cancer, with metastatic disease at presentation or recurrence after surgery. The European Organisation for the Research and Treatment of Cancer Quality of Life Questionnaire Core 30 was used to assess QOL. The association between change in time-dependent QOL (worsening by 10-point or not) and time to treatment alteration was assessed using the Cox regression models controlling for patient characteristics (age, liver metastasis, hormone status, and treatment regimen) and baseline QOL.

**Results:**

Worsening physical functioning, global health status, and dyspnea were significantly associated with treatment discontinuation. Worsening role functioning, global health status, and fatigue were significantly associated with dose reduction. The threshold for defining worsening did not have a significant impact on the relationship.

**Conclusion:**

Changes in QOL are associated with the probability of treatment alteration among metastatic breast cancer patients. Physical functioning, role functioning, global health status, dyspnea, and fatigue should be prioritized for symptom management in patients with metastatic breast cancer.

**Supplementary information:**

The online version contains supplementary material available at 10.1007/s00520-022-07283-0.

## Introduction

Treatment alteration (e.g., treatment discontinuation and dose reduction) in breast cancer treatment is associated with a risk of reduced survival [1, 2]. In metastatic breast cancer patients, dose reduction in taxane-based and anthracycline-based chemotherapy [3] and targeted therapy [4] reduces the progression-free survival (PFS) or overall survival (OS) outcomes. Although patient characteristics (e.g., comorbidities) may confound those associations [5], decreasing treatment alteration may contribute to prolonging survival for metastatic breast cancer. To decrease treatment alteration, monitoring patient’s health conditions, or health-related quality of life (QOL), via patient-reported outcomes (PRO) may be helpful [6]. Therefore, studies have examined the relationship between the deterioration of QOL and treatment alteration [7–9].

However, few studies have provided evidence on the association between QOL and alterations in metastatic breast cancer. The retrospective observational study in the USA [10] showed that moderate or severe patient-reported symptoms were significantly associated with early treatment discontinuation. Nevertheless, this study had several limitations. The number of surveys varied between patients; thus, the symptom burden of patients who completed fewer surveys may have been underassessed. Moreover, they did not assess whether a change in QOL is related to treatment alteration, although analysis focused on changes in QOL over time might provide additional evidence [11]. Analysis of studies in which QOL was assessed at fixed intervals, including baseline (i.e., before initiation of chemotherapy), can provide a more accurate association between (change in) QOL and treatment alteration.

This study aimed to examine the relationship between changes in QOL and treatment alteration (treatment discontinuation and dose reduction) of first-line chemotherapy for metastatic breast cancer, using data obtained from randomized trials. The secondary aim was to examine the relationship between changes in QOL and survival outcomes (i.e., PFS and OS).

## Methods

### Data source and patients

Data from the SELECT BC [12] and SELECT BC-CONFIRM [13] studies were combined. These studies included patients who were diagnosed with human epidermal growth factor receptor 2-negative and endocrine treatment-resistant breast cancer, with metastatic disease at presentation or recurrence after surgery. Both studies were phase 3 randomized clinical trials with QOL assessed as a secondary outcome; due to the limitations of resources (e.g., availability of clinical research coordinators), the assessment of QOL was conducted in only a subset of institutions. The SELECT BC study randomized patients to S-1 and taxane treatments (docetaxel or paclitaxel), while the SELECT BC-CONFIRM study randomized patients to S-1 and anthracycline treatments (doxorubicin or epirubicin). Written informed consent for secondary use of the data was obtained from all the patients.

### Endpoints

Two endpoints related to the time to treatment alteration were considered: time to treatment discontinuation and time to dose reduction. In the trials, treatment was continued until tumor progression or unacceptable toxic effects (e.g., grade ≥ 3 non-hematological adverse events in the Common Terminology Criteria for Adverse Events [14]). After discontinuing the treatment, second-line treatment including treatment switching was allowed within the study protocol. The full criteria for dose reduction are available in the supplementary material. To differentiate treatment discontinuation due to disease progression from that due to other reasons, we censored patients who withdrew from treatments owing to disease progression at the time, similar to previous study [10]. The secondary endpoints were PFS and OS. All endpoints started from the time of randomization.

### Independent variables

The European Organisation for the Research and Treatment of Cancer Quality of Life Questionnaire Core 30 (EORTC QLQ-C30) [15] is one of the most widely used questionnaires for assessing QOL among patients with cancer. It comprises five functional scales (physical, role, cognitive, emotional, and social), a global health status/QOL scale, and nine three symptom scales (fatigue, pain, nausea and vomiting, dyspnea, insomnia, appetite loss, constipation, diarrhea, and financial difficulties). Each scale is converted into a 0–100 scale, in compliance with the scoring manual [16]. A lower score for a functional scale indicates a lower level of functioning (unhealthy level of functioning), and a lower score for the global health status/QOL indicates a lower QOL. Meanwhile, a higher score for a symptom scale or item indicates a higher level of symptoms.

In the SELECT BC study, the questionnaire was administered before the start of treatment and at 3, 6, and 12 months after the initiation of protocol treatment. In the SELECT BC-CONFIRM study, the questionnaire was administered before the start of treatment and at 2, 4, 6, 8, 10, and 12 months after the initiation of protocol treatment. Other variables included age (continuous), liver metastasis (yes or no), hormone receptor status (positive, negative, or unknown), and treatment regimen (taxane-based, anthracycline-based, or S-1).

### Statistical analyses

The patient characteristics and endpoints are presented as the mean and standard deviation for continuous variables and as the number and proportion for categorical variables. Cox regression models with time-dependent QOL were used to assess the relationship of QOL with time to treatment alteration (treatment discontinuation and dose reduction) and survival (PFS and OS). For each scale, the time-dependent QOL can be assessed in several ways: (1) change from baseline, (2) change from baseline categorized such as worsened or not, (3) recent QOL, and (4) recent QOL categorized such as good or bad condition. We focused on the second method (i.e., the change from baseline categorized as worsened or not) because our interests were in the change in QOL from baseline (i.e., before starting chemotherapy) and binary assessment is clinically useful. We used a 10-point threshold to define worsening [17]. For example, if the score of a patient’s symptom scale increased by 10 points, the patient was categorized as worsened.

To assess the association between time-dependent QOL (i.e., change from baseline categorized as worsened or not) additional to the baseline QOL, patient characteristics (age, liver metastasis, hormone status, and treatment regimen) and baseline QOL were controlled in the Cox regression models. Model fit was assessed using the Akaike’s information criteria (AIC). Given the exploratory nature of this study, we did not consider multiple tests. Missing values were not imputed, which correspond to the last observation carried forward method for the change in QOL. All analyses were conducted using SAS (version 9.4; SAS Institute Inc., Cary, NC, USA). Statistical significance was set at *p* < 0.05.

## Results

### Baseline patient characteristics

A total of 543 patients with available baseline QOL scores were included in the analysis. There were no significant differences in baseline characteristics between the patients who were assessed QOL and other patients [12, 18]. Among them, 291 patients belonged to the S-1 group (210 patients from SELECT BC and 81 patients from SELECT BC-CONFIRM), 75 patients belonged to the anthracycline group, and 177 patients belonged to the taxane group. The baseline patient characteristics are shown in Table 1. The median patient age was 59 years, and approximately one-third of the patients had liver metastasis.

**Table 1:** Baseline patient characteristics

		*N*	%
Participated study			
SELECT BC		387	71.3
SELECT BC-CONFIRM		156	28.7
Treatment			
Anthracycline		75	13.8
S-1		291	53.6
Taxane		177	32.6
Age, years			
≤ 50		131	24.1
50–60		184	33.9
60–70		181	33.3
> 70		47	8.7
Median, Q1–Q3		59	51–65
TNM stage			
I		64	11.8
II		242	44.6
III		96	17.7
IV		116	21.4
Unknown		25	4.6
Estrogen receptor			
Positive		388	71.5
Negative		138	25.4
Unknown		17	3.1
History of surgery			
Yes		107	19.7
No		436	80.3
Liver metastasis			
Yes		201	37.0
No		342	63.0

Q1, 25th percentile; Q3, 75th percentile

### Distribution of outcomes and QOL scores

Table 2 shows the summarization of time to event outcomes, and the Kaplan–Meier curves are shown in Supplementary Fig. 1. Treatment discontinuation and dose reduction were observed in 12.2% and 19.9% of the patients, respectively. Almost all treatment alteration events (treatment discontinuation, 100%; dose reduction 95.4%) were observed within a year, that is, within the QOL assessment period. Approximately 50% of PFS events occurred after 1 year, and most OS events were observed after the last assessment of QOL. The primary reason for treatment discontinuation was adverse events (74.2%). The distribution of QOL scores stratified by time point (baseline, 6, and 12 months) is shown in Table 3. The overall response rate was greater than 70%.

**Table 2:** Distribution of treatment alteration and survival outcomes

	Number of events		Time-to-event (months)				
	*N*	%	P5	P25	P50	P75	P95
Treatment discontinuation	66	12.2	0.6	1.2	2.6	3.8	5.4
Dose reduction	108	19.9	1.1	1.5	2.8	6.1	11.7
Progression-free survival	487	89.7	1.9	5.6	10.4	17.9	39.9
Overall survival	380	70.0	4.4	14.3	26.8	36.8	51.4

*P* percentile

**Table 3:** Quality of life scores at baseline, 6, and 12 months after the initiation of chemotherapy

	Baseline			6 months					12 months				
				Raw value			Change from baseline		Raw value			Change from baseline	
	*N*	Mean	SD	*N*	Mean	SD	Mean	SD	*N*	Mean	SD	Mean	SD
PF	543	82.2	19.2	398	80.5	17.2	− 3.0	16.4	318	80.1	19.1	− 4.5	18.1
RF	542	80.9	24.8	397	77.7	24.4	− 4.0	25.5	317	77.5	26.3	− 5.8	28.0
EF	543	72.7	20.2	398	82.6	18.1	9.8	21.1	318	82.1	18.4	9.0	20.9
CF	542	80.0	20.2	398	78.1	19.9	− 2.0	21.2	318	78.5	19.7	− 1.9	20.6
SF	541	81.4	23.8	397	81.4	23.6	− 0.3	26.2	317	82.6	23.5	0.1	26.9
QL	543	59.4	23.1	398	59.5	24.3	0.4	26.4	318	60.4	24.1	− 0.6	24.9
FA	543	31.7	23.2	398	34.6	23.2	2.7	22.8	318	34.7	23.7	3.6	23.7
NV	543	5.2	15.3	397	6.3	14.7	1.3	19.0	318	6.2	14.8	1.7	20.4
PA	543	24.7	24.4	397	22.2	24.8	− 2.1	27.1	318	20.5	23.6	− 1.2	25.8
DY	543	18.5	25.5	397	20.1	23.5	2.1	26.6	318	19.7	22.4	2.2	26.8
SL	542	23.2	27.2	396	20.9	26.6	− 1.6	28.9	317	20.4	24.7	− 1.4	28.0
AP	540	18.6	26.9	396	23.2	27.7	4.7	31.6	315	22.2	27.0	5.7	32.7
CO	543	15.0	23.4	398	20.9	24.8	7.1	28.0	317	19.0	24.0	4.7	27.6
DI	539	7.1	16.5	391	12.2	20.4	4.8	22.9	314	10.1	18.7	3.4	22.0
FI	535	26.9	31.1	394	26.6	29.1	− 0.8	27.9	313	24.5	29.8	− 2.6	32.5

*AP* appetite loss, *CF* cognitive function, *CO* constipation, *DI* diarrhea, *DY* dyspnea, *EF* emotional function, *FA* fatigue, *FI* financial impact, *NV* nausea and vomiting, *PA* pain, *PF* physical function, *QL* global quality of life, *RF* role function, *SD* standard deviation, *SF* social function, *SL* insomnia

### Relationship of change in QOL and treatment alteration

**Fig. 1:**
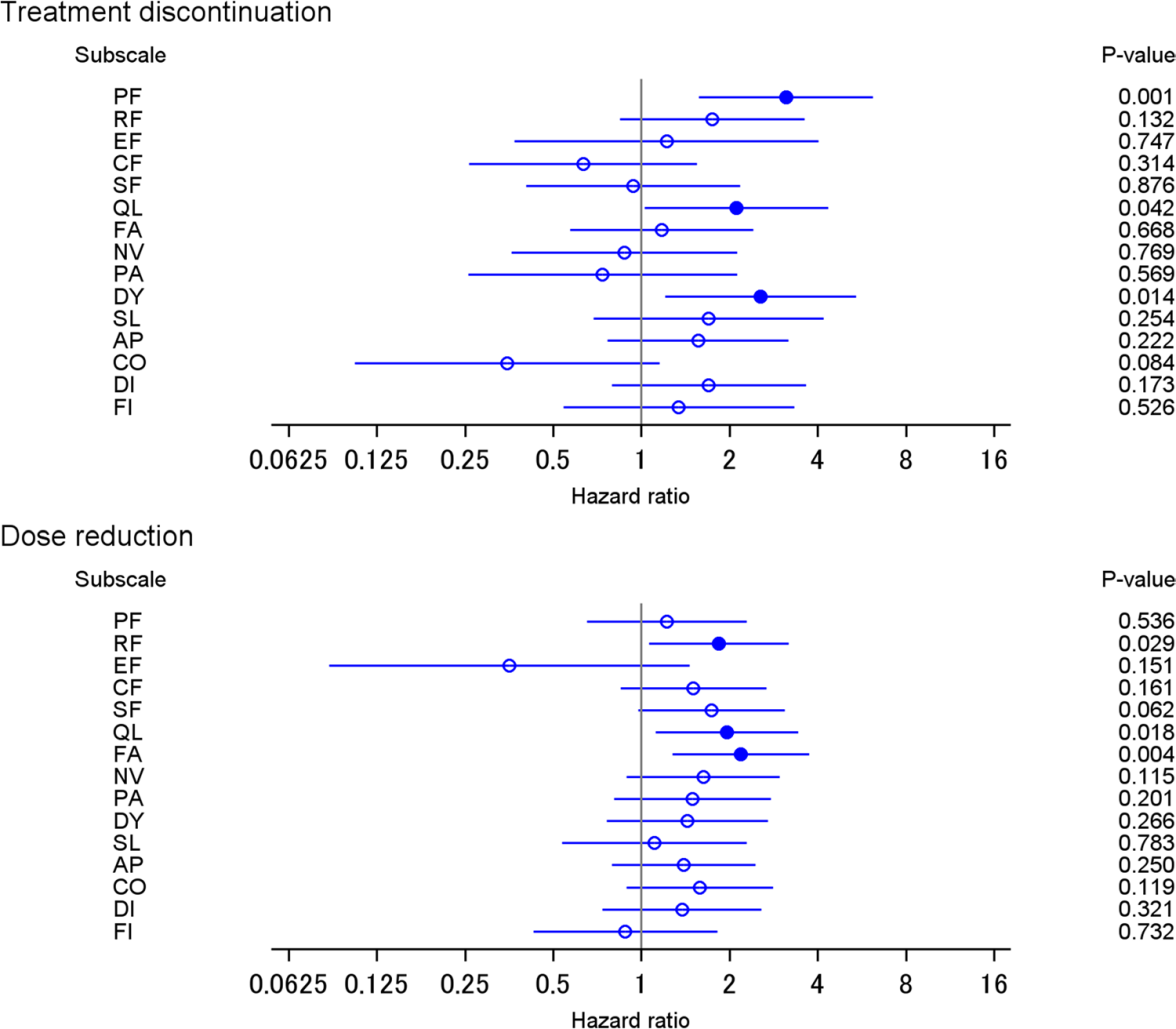
Association of change in quality of life with treatment discontinuation and dose reduction. Significant hazard ratios (i.e., with *P* < 0.05) are shown as filled circles. AP appetite loss, CF cognitive function, CO constipation, DI diarrhea, DY dyspnea, EF emotional function, FA fatigue, FI financial impact, NV nausea and vomiting, PA pain, PF physical function, QL global quality of life, RF role function, SF social function, SL insomnia

Figure 1 presents the estimated hazard ratios for changes in QOL and treatment discontinuation (upper figure) and changes in QOL and dose reduction (lower figure). Among the 15 scales, worsening physical functioning, global health status, and dyspnea was associated with the hazard of treatment discontinuation, after controlling for patient characteristics and baseline score. The estimated hazard ratios were 3.1 (95% confidence interval (CI): 1.6, 6.2) for physical functioning, 2.1 (95% CI: 1.03, 4.3) for global health status, and 2.6 (95% CI: 1.2, 5.4) for dyspnea. Worsening role functioning, global health status, and fatigue were associated with the hazard of dose reduction, after controlling for patient characteristics and baseline scores. The estimated hazard ratios were 1.8 (95% CI: 1.1, 3.2) for role functioning, 2.0 (95% CI: 1.1, 3.4) for global health status, and 2.2 (95% CI: 1.9, 3.7) for fatigue. Some treatment discontinuation events are related to a closely observed worsening physical functioning. Supplementary Fig. 2 is a swimmer plot of 66 patients who discontinued chemotherapy, describing the time lag between worsening physical functioning and treatment discontinuation. Twenty-three patients worsened physical functioning before treatment discontinuation. Within six months from randomization, a total of 57 patients, among 66 patients who discontinued chemotherapy, worsened physical functioning around the treatment discontinuation. On the other hand, only 52% (246/477) of patients worsened physical functioning within the period. Note that the hazard of discontinuing treatment did not change between treatment regimes (hazard ratios were 0.94 and 0.98 in the S-1 and anthracycline groups, respectively, compared to the taxane group, in the analysis of worsening physical functioning).

**Fig. 2:**
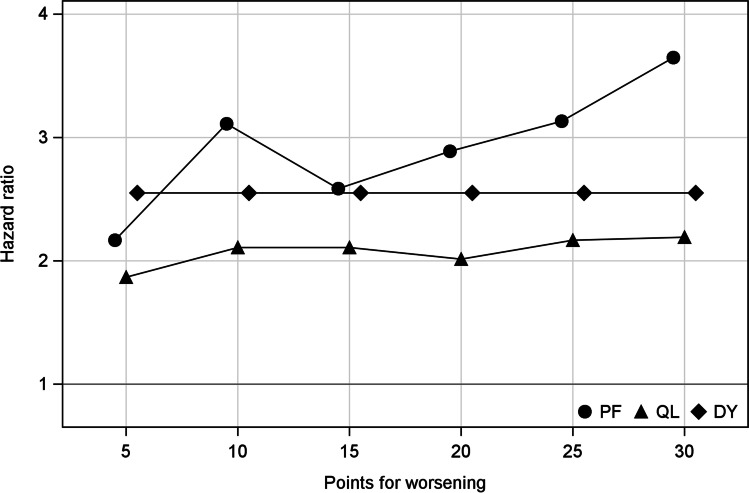
Relationship of the threshold for worsening quality of life and hazard ratios for treatment discontinuation. DY dyspnea, PF physical function, QL global quality of life

To further describe the magnitude of the hazard ratios between changes in QOL and treatment discontinuation, trends in hazard ratios were calculated by adjusting the threshold from 5 to 30 points (Fig. 2). Hazard ratios of the physical functioning and global health status were smaller with a 5-point threshold than with a 10-point threshold, but they did not differ significantly between 10- and 25-point thresholds. Similar analyses for the other scales did not show significant relationships. For physical functioning and global health status, AIC showed the best model fit when using the thresholds of 10-point.

### Relationship of change in QOL with progression-free survival and overall survival

**Fig. 3:**
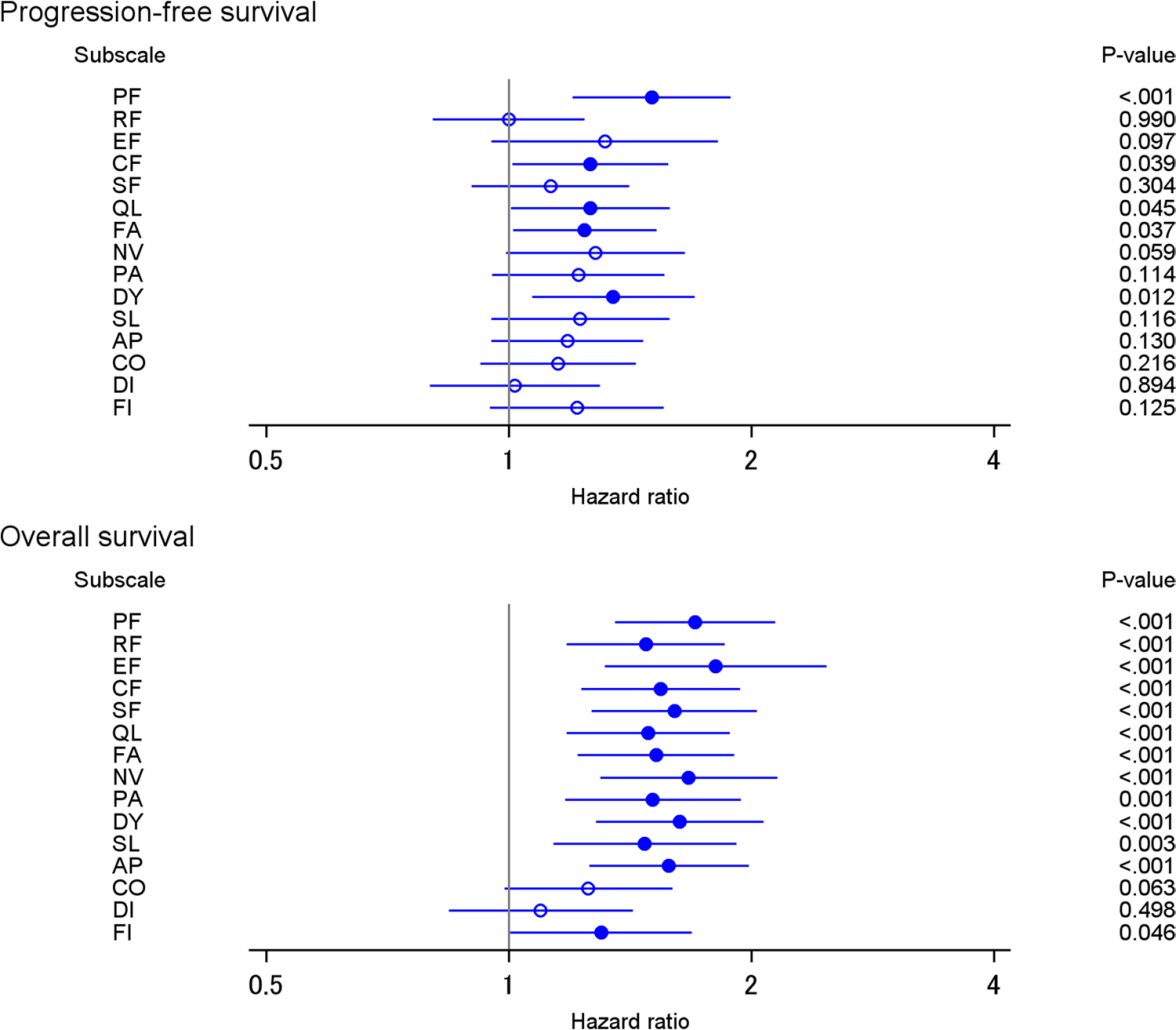
Association of change in the quality of life with progression-free survival and overall survival. Significant hazard ratios (i.e., with *P* < 0.05) are shown as filled circles. AP appetite loss, CF cognitive function, CO constipation, DI diarrhea, DY dyspnea, EF emotional function, FA fatigue, FI financial impact, NV nausea and vomiting, PA pain, PF physical function, QL global quality of life, RF role function, SF social function, SL insomnia

Figure 3 presents the estimated hazard ratios for changes in QOL and PFS (upper figure) and changes in QOL and OS (lower figure). Most scales were consistently associated with PFS and OS. Worsening QOL was related to the hazard of progression and death. Among the 15 scales, physical functioning, cognitive functioning, global health status, fatigue, and dyspnea were significantly associated with PFS. All scales, except constipation and diarrhea, were significantly associated with OS.

## Discussion

This study shows that changes in QOL are associated with time to treatment alteration (treatment discontinuation and dose reduction) and survival (PFS and OS) in patients with metastatic breast cancer, using data from two randomized clinical trials. In the trials, the timing of the QOL assessment is standardized for all patients. QOL was assessed with the EORTC QLQ-C30, which comprises five functional scales, one global health status, and nine symptom scales. Worsening physical functioning, global health status, and dyspnea is significantly associated with the hazard of treatment discontinuation, after controlling for patient characteristics and baseline QOL scores. Further, worsening role functioning, global health status, and fatigue is significantly associated with the hazard of dose reduction, after controlling for patient characteristics and baseline QOL scores. Moreover, changes in many scales are associated with PFS or OS. To the best of our knowledge, this study is the first to show that changes in QOL have additional value for predicting prognosis in metastatic breast cancer patients owing to their association with the probability of treatment alteration.

We demonstrated that changes in functional scales (physical and role functioning), global health status, and symptom scales (dyspnea and fatigue) are associated with treatment discontinuation or dose reduction. Previously, Walker et al. [10] reported that the recent symptom burden was associated with treatment discontinuation. We added two findings to the previous literature. First, functional scales are also associated with time to treatment alteration. Previous studies have shown that functional scales (at baseline) are related to survival among cancer patients [19] or advanced cancer patients [20]. Physical functioning might be more predictive of survival than are other functional scales [20].

Second, we demonstrated changes from baseline provide additional information to the baseline QOL score. Previous studies which analyzed baseline QOL [7–9] may have considered between-patient differences. For example, a higher pain score at baseline may indicate not only true pain, but also a tendency of the patient to overrate the pain. Compared to these analyses, analyses that use changes in QOL from baseline attempt to cancel the between-patient change by subtracting the baseline score. Hence, the analysis may determine the relationship between the true change in QOL in a patient and outcomes. In our analysis, we controlled for baseline QOL to account for residual between-patient differences by using regression models. The results showed that changes in QOL provide additional information to the baseline QOL score that can be useful for determining treatment alteration and survival. Although change in QOL may be perceived to have lower power than baseline QOL or recent QOL, in some settings, only recent QOL, but not change in QOL, is associated with survival [21]. Therefore, power comparison between these methods might be informative.

To define the worsening QOL, we used a 10-point threshold in the analysis. Other definitions such as using the minimal important difference to define worsening [17] are alternatives. However, in our analysis of the change in hazard ratios by moving the thresholds, we found that there were small changes in the estimates of hazard ratios from 10- to 30-point thresholds. Thus, we believe that our finding that some scales are associated with time to treatment alteration is robust. Two points are worth noting. First, only four distinct values (i.e., 0, 33.3, 66.7, and 100) can be taken for single symptom items (dyspnea, insomnia, appetite loss, constipation, diarrhea, and financial difficulties), shown as the horizontal plot for dyspnea in Fig. 2. Therefore, small changes are not important in some scales. Second, the minimal important difference should be distinguished as between-group change and within-group change [22]. Thus, when choosing to use minimal important difference as a definition of worsening, one should carefully check whether it was calculated using appropriate statistical methods, including anchor characteristics [23].

The scales selected in this study should be prioritized for symptom monitoring. The mainstay of treatment for metastatic breast cancer is chemotherapy, which is accompanied by adverse events. Therefore, continuing treatment only to prolong survival may lead to worsened QOL and increased medical costs. Doctors tend to underestimate patient symptoms [24]. However, patient monitoring is becoming increasingly popular with the progress of Internet of Things. Although we used paper-based PRO in the clinical trials, electronic PRO will enable medical practitioners to grasp the patients’ health status quickly and accurately. The effectiveness of electronic PRO is improving. For example, the symptom burden is reported to be reduced with the support of an interactive application, which enables early identification and management of symptoms and facilitates interaction with healthcare professionals [25].

Changes in physical functioning, cognitive functioning, global health status, fatigue, and dyspnea were significantly associated with PFS. Some of the QOL scales (physical functioning, global health status, and dyspnea) were also associated with treatment discontinuation. Interestingly, although progression was excluded from the definition of treatment discontinuation, several QOL scales were associated with both treatment discontinuation and PFS. Many previous studies have found that a range of QOL scales are associated with survival in cancer patients [11, 19, 26, 27]. Contrarily, evidence on the relationship between changes in QOL and survival is rare. This study provides evidence of the influence of changes in QOL on cancer survival.

The strength of our study includes the fact that we controlled for baseline QOL to account for residual between-patient differences (baseline QOL) and revealed the additional predictive value of changes in QOL. Despite the strengths, this study also had several limitations. First, the patients with metastatic breast cancer included in this study were selected to participate in the trials, and they had ≤ 1 performance status scores of Eastern Cooperative Oncology Group [28]. Second, we used the definition of dose reduction noted in the protocol, not the more commonly used relative dose intensity definition [29]. The trials were pragmatic, and the chemotherapy dose was decided by the attending physician. Therefore, we selected a simple definition of dose reduction rather than a relative dose compared to a standard dose variable between patients. Lastly, there was a significant time gap between the OS and the time to the last assessment of QOL (Supplementary Fig. 1), which was only assessed for up to 1 year. Future research is needed to establish the relationship between QOL and OS within a short period of time, possibly using electronic PRO.

## Conclusions

Worsening physical functioning, global health status, and dyspnea are significantly associated with treatment discontinuation, after controlling for patient characteristics and baseline QOL. Similarly, worsening role functioning, global health status, and fatigue is significantly associated with dose reduction. These scales should be prioritized for symptom monitoring in patients with metastatic breast cancer.

## Supplementary information

Below is the link to the electronic supplementary material.

Supplementary file1 (PDF 277 KB)

## Data Availability

The datasets during and/or analyzed during the current study are available from the corresponding author on reasonable request.

## Code availability

The SAS codes generated during the current study are available from the corresponding author on reasonable request.
