# Supplementary material for: Association of change in health-related quality of life and treatment discontinuation in metastatic breast cancer: a post hoc, exploratory analysis of two randomized clinical trials
Source: Support Care Cancer. 2022 Jul 20;30(10):8367–75. doi: 10.1007/s00520-022-07283-0 (PMC9512887; doi:10.1007/s00520-022-07283-0)
Supplement: Supplementary file 1 — Supplementary file1 (PDF 277 KB) [file 520_2022_7283_MOESM1_ESM.pdf]

Supplementary figures

Supplementary figure 1. Kaplan-Meier curves for time to treatment discontinuation (upper left), time to dose reduction (upper right), progression-free survival (bottom left), and overall survival (bottom right).

Legend for supplementary figure 1: QOL, quality of life.

Supplementary figure 2. Swimmer plot describing the time lag between worsening physical functioning and treatment discontinuation.

Legend for supplementary figure 2: PF, physical functioning.

Supplementary Figure 1

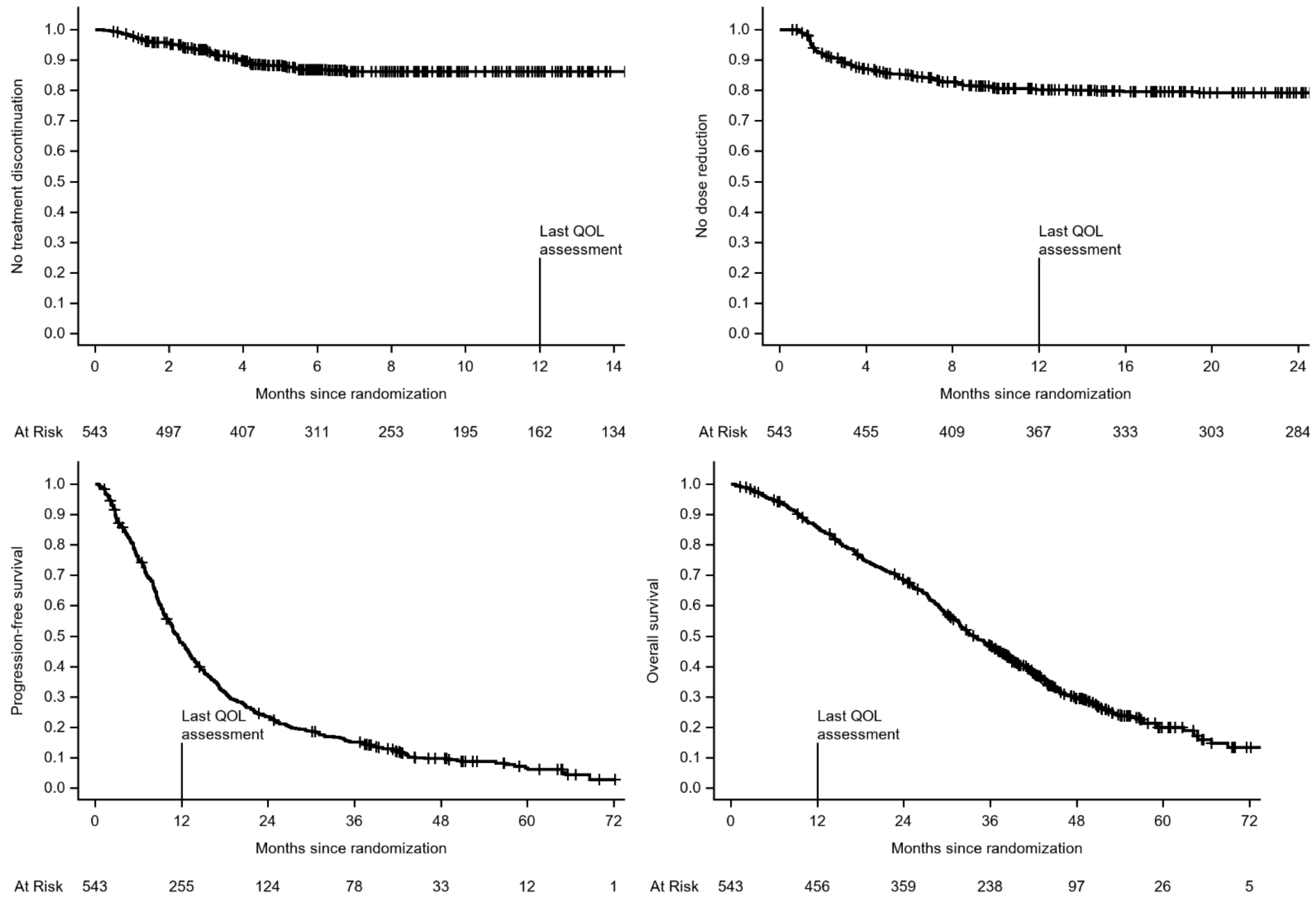

Supplementary Figure 2

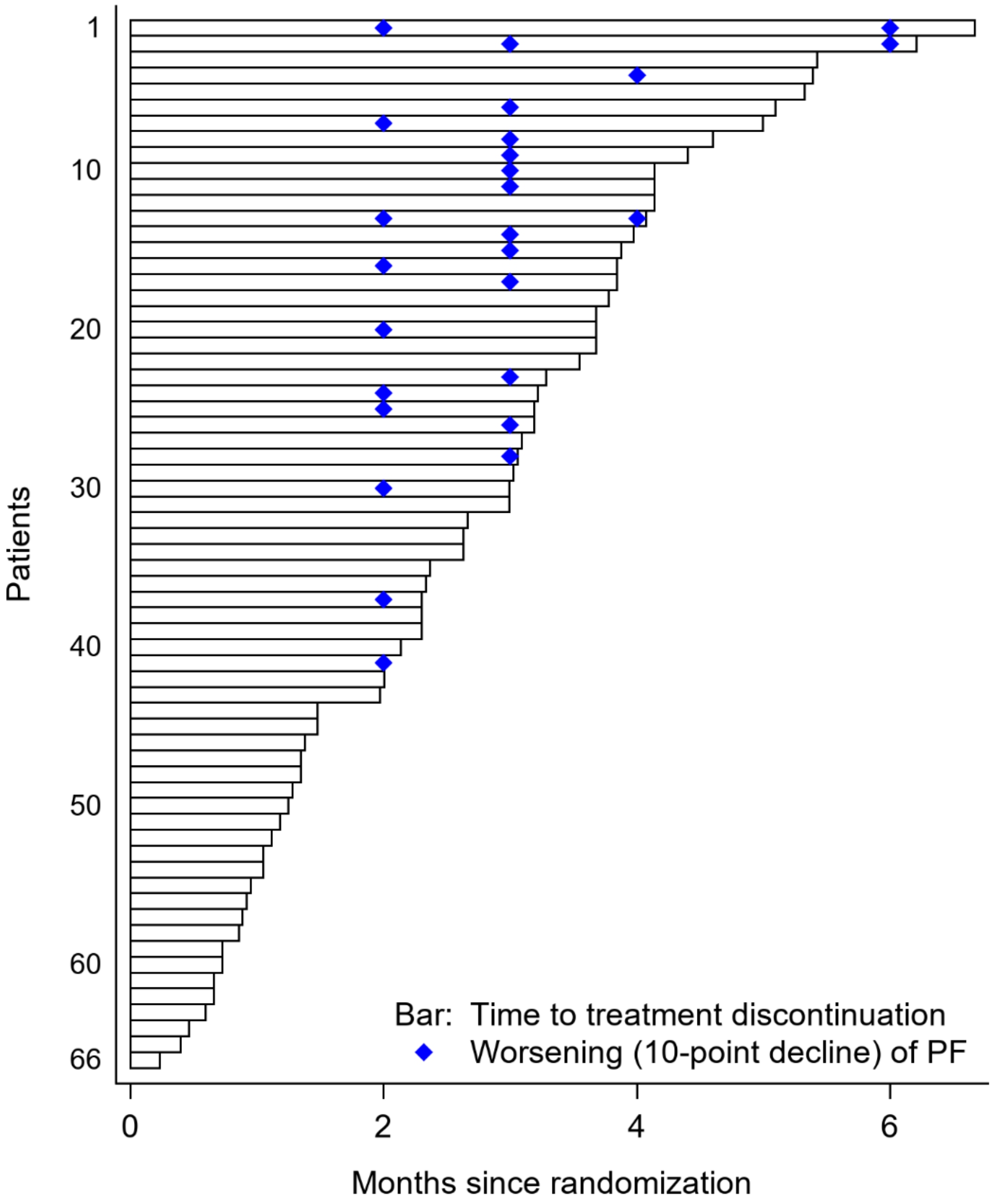

Supplementary Material: Dose reduction criteria

Taxane group

1） Pyrogenic neutropenia of Grade ≥3

2） Platelet count of ≤50,000/mm^3 and subcutaneous hemorrhage

3） Platelet count of ≤25,000/mm^3

4） Platelet count of <100,000/mm^3 on day prior to or day of course commencement

5） Grade 3 severity of any of the following non-hematological toxicities: weight decrease, weight gain, diarrhea, nausea/vomiting, neuropathy-motor, neuropathy-cognitive, joint pain, myalgia, and fatigue.

Anthracycline group

1) Febrile neutropenia of Grade ≥3

2) Platelet count ≤ 50,000 /mm^3 and subcutaneous hemorrhage

3) Platelet count ≤ 25,000 /mm^3

4) Platelet count on the day of administration or on the day before < 100,000 /mm^3

5) Any of the following non-hematological Grade 3 toxicities: weight loss, weight gain, diarrhea, nausea and vomiting, and fatigue

S-1 group (SELECT BC study)

(1) Grade ≥4 hematological toxicities (neutrophils, hemoglobin, and platelets)

(2) Grade ≥3 of any of the following non-hematological toxicities:

Non-hematological toxicities: adverse events outside the CTCAE v3.0 “blood/bone-marrow” classification

S-1 group (SELECT BC CONFIRM study)

(1) Grade ≥3 of any hematological toxicities (neutrophils, hemoglobin, platelets).

(2) Grade ≥3 of any of the following non-hematological toxicities:

Non-hematological toxicities: adverse events outside the CTCAE v3.0 “blood/bone-marrow” classification
